# Cytokines and chemokines multiplex analysis in patients with low disease activity rheumatoid arthritis

**DOI:** 10.1007/s00296-022-05103-6

**Published:** 2022-02-18

**Authors:** Maria Skrzypkowska, Mariusz Stasiak, Justyna Sakowska, Joanna Chmiel, Agata Maciejewska, Adam Buciński, Bartosz Słomiński, Piotr Trzonkowski, Piotr Łuczkiewicz

**Affiliations:** 1 Department of Medical Immunology, Faculty of Medicine, Medical University of Gdańsk, Dębinki 1, 80-210 Gdańsk, Poland; 2 Second Clinic of Orthopaedics and Kinetic Organ Traumatology, Faculty of Medicine, Medical University of Gdańsk, Gdańsk, Poland; 3 Department of Biopharmacy, Nicolaus Copernicus University in Toruń, Collegium Medicum in Bydgoszcz, Bydgoszcz, Poland

**Keywords:** Rheumatoid arthritis, Osteoarthritis, IL-2, Eotaxin/CCL11, MDC/CCL22, GM-CSF, IL-10, IL-1RA

## Abstract

Rheumatoid arthritis is a severe chronic autoimmune disorder that results from pathological activation of immune cells and altered cytokine/chemokine network. The aim of our study was to evaluate concentrations of chosen cytokines and chemokines in blood sera and synovial fluid samples isolated from low disease activity rheumatoid arthritis (RA) patients and osteoarthritis (OA) sufferers. Blood sera and synovial fluid samples have been obtained from 24 OA and 14 RA patients. Cytokines/chemokines levels have been determined using a Milliplex^®^ Map 38-plex human cytokine/chemokine magnetic bead-based panel (Merck Millipore, Germany) and Luminex^®^ MAGPIX^®^ platform (Luminex USA). Low disease activity RA patients showed altered concentration of numerous cytokine/chemokine when compared to OA controls—they were characterized by, inter alia, increased: eotaxin/CCL11 (*p* = 0.037), GRO/CXCL1 (*p* = 0.037), IL-2 (*p* = 0.013), IL-4 (*p* = 0.017), IL-7 (*p* = 0.003), IL-8 (*p* = 0.0007) and GM-CSF (*p* = 0.037) serum levels, whilst MDC/CCL22 concentration was decreased in this group (*p* = 0.034). Eotaxin/CCL11 (*p* = 0.001), GRO/CXCL1 (*p* = 0.041), IL-10 (*p* = 0.003), GM-CSF (*p* = 0.01), IL-1RA (*p* = 0.0005) and VEGF (*p* = 0.01) concentrations in synovial fluid of RA females were also increased. Even with low disease activity score, RA patients exhibited increased concentrations of cytokines with pro- and anti-inflammatory activities, as well as numerous chemokines, growth factors and regulators of angiogenesis. Surprisingly, RA subjects also shown decreased concentration of CCL22 chemokine. The attempt to restore cytokine balance and tolerogenic environment is ineffective in RA sufferers even with good disease management. Distinguished factors could serve as possible indicators of disease progression even in low disease activity patients.

## Introduction

Rheumatoid arthritis (RA) is a severe chronic autoimmune disorder that is believed to affect approximately 1% of world’s population. It develops as a consequence of lack of proper inflammation resolution that lasts throughout patients’ lifetime [1]. Despite the exact cause of RA being still enigmatic, it has been acknowledged that genetic factors play pivotal role in RA onset and progression. Specific and non-specific leukocytes that migrate into joints interact with synovial tissue-resident cells as well as each other, thus creating continual highly destructive synovitis [2, 3]. This sophisticated communication between cells is heavily dependent on cytokines and chemokines. Numerous cytokines have been indicated as playing pivotal role in RA including: TNF (tumor necrosis factor), IL-1 (interleukin 1), IL-6 (interleukin 6), or IL-12 (interleukin 12) to name just a few [4]. Cytokines affect: phenotype, migration, activity and survival of cells in joints and lymphoid tissues [5] and their secretion leads to induction of different pathways and expression of various genes resulting in tissue destruction. The irreversible joint injury, mobility attenuation and disability then follow [4]. The unraveling of complex cytokine network is hindered by the dependence of their physiological effects on the stage of RA or tissue microenvironment. The disease mainly damages synovial membranes, cartilages and bones, but it also causes systemic threat by putting distress on other organs and tissues, as well [1]. RA takes a toll on cardiovascular system, psychological health or risk of cancer development. Despite successful TNF- or IL-6-targeted biological therapies, the frequency of refractory RA creates growing need to better understand cytokine hierarchy and seek for additional cytokine-focused therapeutic approaches [5].

Osteoarthritis (OA) is the most frequent chronic joint disease [6] and the most common reason for total hip and knee replacement. It is characterized by progressive degeneration of articular cartilage and increase in bone density. OA patients suffer from painful joints, functional impairment and disability [7]. With rather enigmatic etiology, OA was first recognized as a non-inflammatory joint dysfunction induced by mechanical stress or age-related degenerations [8]. However, recent studies suggested revision of OA to a low-grade chronic inflammation within leukocyte-infiltrated synovial tissue [9].

Bahlas et al. have proven Luminex technology to be useful tool to evaluate: cytokines, chemokines and growth factors in blood sera samples of RA sufferers [10]. The purpose of our analysis was to compare concentrations of these factors in blood sera as well as synovial fluid samples isolated from low disease activity RA and OA sufferers.

## Materials and methods

### Patients

Twenty-four patients diagnosed with OA and fourteen individuals with recognized RA have been included into our study. All participants have fulfilled American College of Rheumatology (ACR) criteria for RA [11] and OA [12] and have been scheduled to undergo arthroplasty of knee joint in the 2nd Division of Orthopaedics and Kinetic Organ Traumatology of University Clinical Centre in Gdańsk. Information received from each patient included: age, smoking history, body mass index (BMI), history of: hypertension, diabetes, cancer, cardiovascular diseases, autoimmune diseases, infectious diseases and previous surgical procedures and current medication. The inclusion criteria were as follows: age between 18 and 79 years, severity of knee OA in Kellgren–Lawrence score of 4, Disease Activity Score 28 ≤ 3.2 in RA group and agreement to participate in the study. The exclusion criteria were as follows: patient affected by autoimmune diseases other than RA, intraarticular steroid injection in the last 6 month before surgery, and biological treatment in patient with RA. Written informed consent was obtained from all participants. The study was approved by the Ethics Committee of Medical University of Gdańsk (consent no NKBBN/642/2018 received on December 10, 2018) and our investigation was carried out in accordance with the Code of 8 Ethics of the World Medical Association (Declaration of Helsinki) for experiments on human subjects.

### Samples collection and preparation

Venous blood was collected into clot activator-containing tubes and allowed to clot for 30 min followed by centrifugation for 15 min at 1000*g*. Aliquoted serum samples were stored at − 80 °C for further analyses. Synovial fluid was collected into sterile tubes by joint puncture during the arthroplasty of knee joint procedure—sample was obtained into sterile syringe through small 1–2 cm longitudinal mid-axial incision made approximately 2–4 cm above superior pole of patella. Samples of synovial fluid were centrifuged for 10 min at 300 g to remove debris and stored at – 80 °C until needed.

### Determination of cytokines and chemokines

Serum and synovial fluid cytokines and chemokines concentrations have been determined using a Milliplex® Map human cytokine/chemokine magnetic bead-based panel (Merck Millipore, Germany) according to the manufacturer’s instructions. Chosen factors have been analysed on the Luminex® MAGPIX® platform (Luminex USA). The 38-plex assay has allowed us to evaluate the following factors: interleukin 1α (IL-1α), interleukin 1β (IL-1β), interleukin 2 (IL-2), interleukin 3 (IL-3), interleukin 5 (IL-5), interleukin 7 (IL-7), interleukin 8 (IL-8), interleukin 9 (IL-9), interleukin 12p40 (IL-12p40), interleukin 12p70 (IL-12p70), interleukin 15 (IL-15), interleukin 17A (IL-17A), epidermal growth factor (EGF), fibroblast growth factor 2 (FGF-2), transforming growth factor α (TGF-α), granulocyte-colony-stimulating factor (G-CSF), eotaxin/CCL11, FMS-like tyrosine kinase 3 ligand (Flt-3L), granulocyte–macrophage colony-stimulating factor (GM-CSF), fractalkine/CX3CL1, interferon α2 (IFN-α2), interferon γ (IFN-γ), growth-regulated alpha protein (GRO)/CXCL1, monocyte-chemotactic protein 3 (MCP3)/CCL7, macrophage-derived chemokine (MDC)/CCL22, soluble CD40 ligand (sCD40L), inducible protein 10 (IP-10)/CXCL10, monocyte-chemotactic protein 1 (MCP-1)/CCL2, macrophage inflammatory protein-1α (MIP-1α)/CCL3, macrophage inflammatory protein-1β (MIP-1ß)/CCL4, tumor necrosis factor α (TNF-α), tumor necrosis factor β (TNF-β) and vascular endothelial growth factor (VEGF). Simultaneously, we have evaluated the following anti-inflammatory factors: interleukin 1 receptor antagonist (IL-1RA), interleukin 4 (IL-4), interleukin 6 (IL-6), interleukin 10 (IL-10) and interleukin 13 (IL-13).

### Statistics

Cytokines and chemokines detected in less than 50% of samples have been excluded from analysis. The results were analysed using the Statistica, version 12.0 (StatSoft Inc, USA). Due to small sample size, variables have been presented as median with minimum—maximum and evaluated using the Mann–Whitney *U* test. Fisher’s exact test was applied for dichotomous variables concerning clinical characteristics of participants. Values of *p* < 0.05 were considered statistically significant.

## Results

### Clinical characteristics of analysed groups

OA group was older (65 vs 50; *p* = 0.0006) and had higher BMI values (30 vs 24.5; *p* = 0.00001) when compared to RA subjects did not reach statistical significance (*p* = 0.068 and 0.069 respectively). OA group developed arterial hypertension (*p* = 0.0003) and hyperlipidemia (*p* = 0.04) and, consequently, were treated with *β* blockers (*p* = 0.004) and angiotensin-converting enzyme inhibitors (ACEI) (*p* = 0.004) more frequently than RA sufferers. The majority of RA patients were treated disease-modifying anti-rheumatic drugs (DMARDs) (*p* = 0.00004) and steroids (*p* = 0.00001) (Table 1).

**Table 1:** Characteristics of osteoarthritis and rheumatoid arthritis patients

Characteristic	OA [24]	RA [14]	*p*
Gender (f/m)	19/5	12/2	0.48
Age (years)	65 (56–74)	50 (39–71)	0.0006*
BMI (kg/m^2^)	30 (24–41)	24.5 (19–32)	0.00001*
Smoking (*n*)	5	2	0.48
Osteoporosis (*n*)	3	4	0.22
Diabetes (*n*)	6	1	0.17
AH (*n*)	20	3	0.0003*
Hypothyroidism (*n*)	7	3	0.45
Hyperlipidemia (*n*)	9	1	0.04*
NSAIDs (*n*)	6	3	0.61
DMARDs (*n*)	0	9	0.00004*
Steroids (*n*)	1	11	0.00001*
*β* blockers (*n*)	14	2	0.004*
ACEI (*n*)	14	2	0.004*
ARBs (*n*)	3	0	0.19
Diuretics (*n*)	7	1	0.09

Age and BMI are presented as median and minimal-maximal values; abbreviations: *n* number of subjects, *ACEI* angiotensin-converting-enzyme inhibitors, *AH* arterial hypertension, *ARBs* angiotensin II receptor blockers, *BMI* body mass index, *DMARDs* disease-modifying anti-rheumatic drugs, *NSAIDs* non-steroidal anti-inflammatory drugs; *p* indicates significance between analysed groups; statistically significant differences (*p* < 0.05) between the groups have been highlighted and marked with “*”

### Cytokines and chemokines blood serum levels

Among detected serum-derived factors: TGF-α (*p* = 0.29), IFN-γ (*p* = 0.10), sCD40L (*p* = 0.79), IL-1RA (*p* = 0.17), IP-10/CXCL10 (*p* = 0.22), MCP-1/CCL2 (*p* = 0.26), and TNFα (*p* = 0.37) have exhibited similar levels (Table 2).

**Table 2:** Comparable cytokine and chemokine serum levels in in patients with established osteoarthritis and rheumatoid arthritis

Factor	OA			RA			*p*
	Median	Min–max	95% CI	Median	Min–max	95% CI	
TGF-α (pg/ml)	5.31	1.65–19.34	3.6–6.5	7.1	3.4–24.8	4.0–8.8	0.29
IFN-γ (pg/ml)	3.2	0.0–44.0	7.2–13.1	7.9	0.0–14.8	3.2–7.0	0.10
sCD40L (pg/ml)	8.3	2.1–16.1	2.7–4.9	6.2	2.1–17.9	3.9–9.0	0.79
IL-1RA (pg/ml)	12.3	0.0–58.8	12.6–23.1	23.7	0.0–82.3	16.7–38.3	0.17
IP-10/CXCL10(pg/ml)	177.1	68.1–388.8	50.3–92.0	206.1	84.2–415.3	73.2–162.7	0.22
MCP-1/CCL2 (pg/ml)	485.5	305.6–716.5	84.9–153.2	574.4	317.9–840.6	119.1–264.7	0.26
TNFα (pg/ml)	23.8	16.0–38.9	4.7–8.6	25.2	13.4–36.0	5.3–11.7	0.37

Statistical evaluation of differences was performed using Mann–Whitney *U* test; *p* indicates significance between analysed groups

**Fig. 1:**
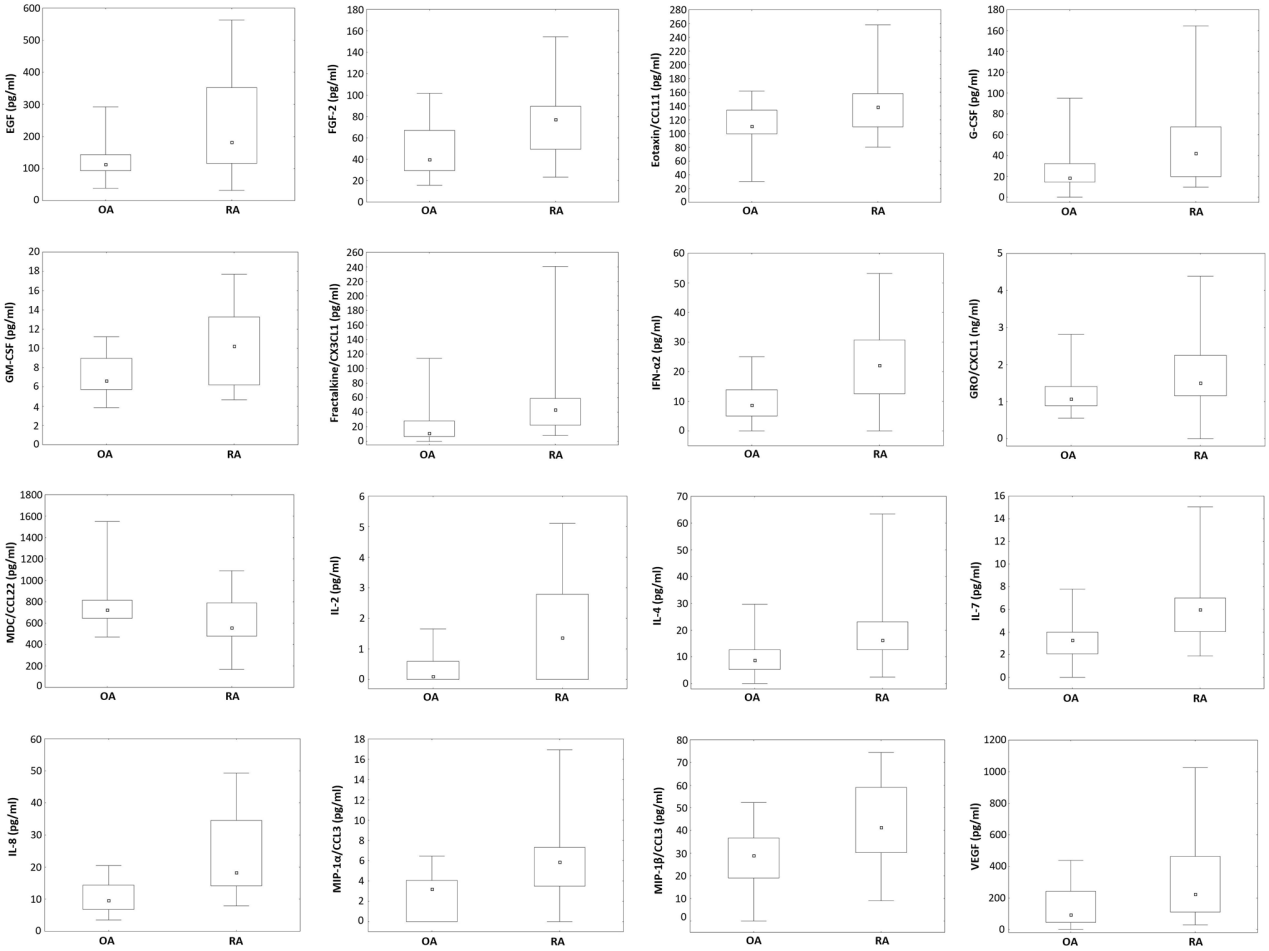
Significantly different cytokine and chemokine serum levels in patients with established osteoarthritis and rheumatoid arthritis. Rheumatoid arthritis is associated with increased serum levels of: EGF (*p* = 0.031) FGF-2 (*p* = 0.017), eotaxin/CCL11 (*p* = 0.037), G-CSF (*p* = 0.014) GM-CSF (*p* = 0.037), fractalkine/CX3CL1 (*p* = 0.002), IFN-α2 (*p* = 0.002), GRO/CXCL1 (*p* = 0.01), IL-2 (*p* = 0.013), IL-4 (*p* = 0.017), IL-7 (*p* = 0.003), IL-8 (*p* = 0.0007), MIP-1-α/CCL3 (*p* = 0.001), MIP-1β/CCL4 (*p* = 0.010) and VEGF (*p* = 0.027). MDC/CCL22 serum levels are lower in RA group (*p* = 0.034). Data are depictured as median with 25–75% percentiles and minimum—maximum and evaluated using the Mann–Whitney U test. Statistical significance was set at *p* < 0.05

The RA group has been characterized by higher serum levels of the following proteins when compared to OA sufferers: EGF (182.5, 31.6–562.6 vs 113.0, 39.0–292.7; *p* = 0.031) FGF-2 (77.0, 23.3–154.5 vs 39.8, 15.7–101.8; *p* = 0.017), eotaxin/CCL11 (138.6, 80.4–258.0 vs 110.7, 30.2–161.6; *p* = 0.037), G-CSF (42.0, 9.5–164.5 vs 18.3, 0.0–95.1; *p* = 0.014) GM-CSF (10.2, 4.7–17.7 vs 6.6, 3.8–11.2; *p* = 0.037), fractalkine/CX3CL1 (43.3, 7.9–240.7 vs 11.0, 0.0–114.2.0; *p* = 0.002), IFN-α2 (22.2, 0.0–53.2 vs 8.7, 0.0–25.0; *p* = 0.002), GRO/CXCL1 (1.5, 0.0–4.4 vs 1.07, 0.55–2.82; *p* = 0.01), IL-2 (1.36, 0.0–5.1 vs 0.1, 0.0–1.7; *p* = 0.013), IL-4 (16.2, 2.4–63.5 vs 8.7, 0.0–29.7; *p* = 0.017), IL-7 (6.0, 1.9–15.1 vs 3.3, 0.0–7.8; *p* = 0.003), IL-8 (18.3, 7.9–49.3 vs 9.6, 3.5–20.5; *p* = 0.0007), MIP-1-α/CCL3 (5.9, 0.0–16.9 vs 3.2, 0.0–6.5; *p* = 0.001), MIP-1β/CCL4 (41.3, 8.9–74.4 vs 28.9, 0.0–52.4; *p* = 0.010) and VEGF (223.5, 30.1–1025 vs 92.9, 0.0–437.8; *p* = 0.027). Simultaneously, MDC/CCL22 serum levels were lower in RA group (556.2, 167.7–1090.7 vs 724.2, 470.5–1549.0; *p* = 0.034) (Fig. 1).

### Cytokines and chemokines’ synovial fluid levels

Within cytokines and chemokines detected in synovial fluid samples, FGF-2 (*p* = 0.84), Flt-3L (*p* = 0.08), MDC/CCL22 (*p* = 0.22), IL-15 (*p* = 0.52), IL-8 (*p* = 0.26), IP-10/CXCL10 (*p* = 0.16), MCP-1/CCL2 (*p* = 0.16) and MIP-1β/CCL4 (*p* = 0.75) and TNFα (*p* = 0.12) have not differed between groups (Table 3).

**Table 3:** Comparable cytokine and chemokine synovial fluid levels in patients with established osteoarthritis and rheumatoid arthritis

Factor	OA			RA			
	Median	Min–max	95% CI	Median	Min–max	95% CI	*p*
FGF-2 (pg/ml)	128.6	0.0–712.7	180–324.8	107.5	0.0–1357.0	279.1–620.2	0.84
Flt-3L (pg/ml)	71.7	0.0–170.4	36.9–66.7	45.4	0.0–130.7	28.8–66.2	0.08
MDC/CCL22 (pg/ml)	160.7	72.4–252.4	36.7–66.2	137.5	68.6–259.8	45.3–100.6	0.22
IL-15 (pg/ml)	17.1	4.7–34.4	5.3–9.6	16.2	3.6–27.7	5.7–13.1	0.52
IL-8 (pg/ml)	17.6	5.6–375.0	72.0–129.9	41.8	5.2–188.6	36.1–80.2	0.26
IP-10/CXCL10 (pg/ml)	530.5	118.9- 1897.0	284.5–513.4	654.8	472.3–1003.5	146.4–325.3	0.16
MCP-1/CCL2 (pg/ml)	619.5	381.5–1351.0	175.1–325.2	1046.6	172.5–1576.0	386.3–858.4	0.16
MIP-1β/CCL4 (pg/ml)	19.0	0.0–48.2	8.9–16.1	20.6	0.0–189.4	35.5–79.0	0.75
TNFα (pg/ml)	6.15	0.0–11.7	2.0–3.6	8.8	0.0–31.3	6.5–14.4	0.12

Statistical evaluation of differences was performed using the Mann–Whitney U test; p indicates significance between analysed groups

**Fig. 2:**
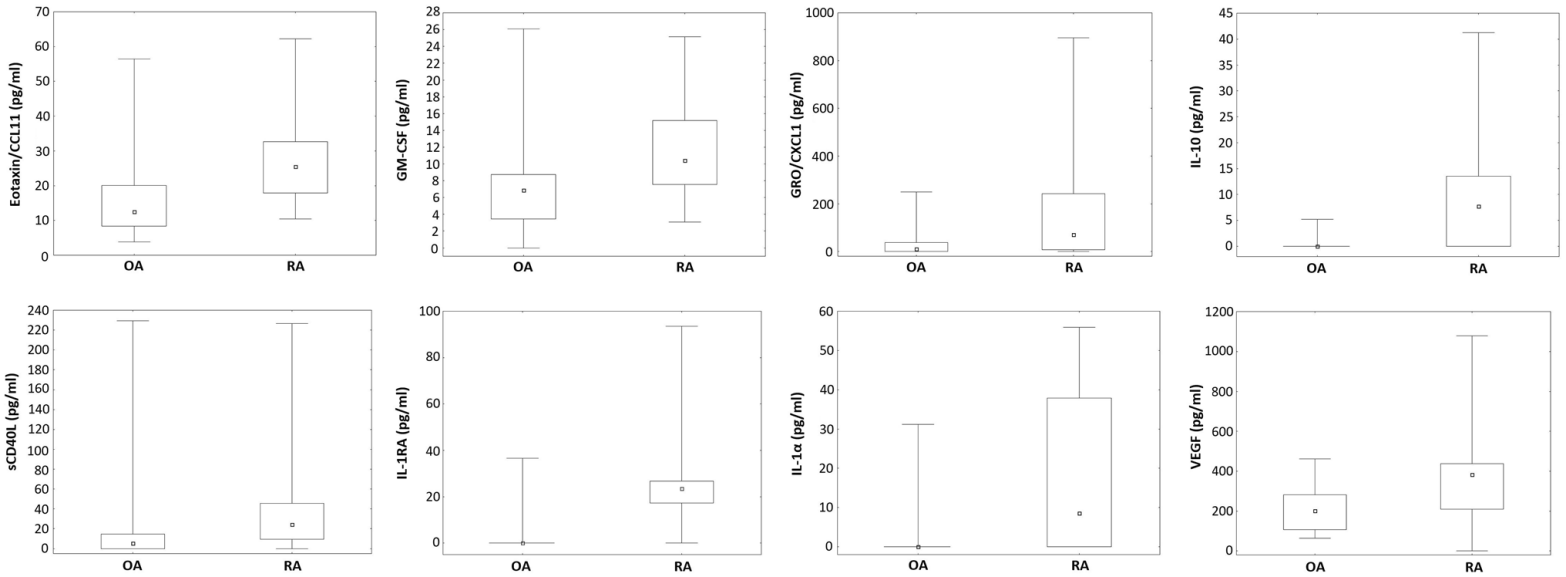
Significantly different cytokine and chemokine synovial fluid levels in patients with established osteoarthritis and rheumatoid arthritis. Rheumatoid arthritis is associated with increased synovial fluid concentrations of: eotaxin/CCL11 (*p* = 0.001), GM-CSF (*p* = 0.01), GRO/CXCL1 (*p* = 0.041), IL-10 (*p* = 0.003), sCD40L (*p* = 0.001), IL-1RA (*p* = 0.0005), IL-1α (*p* = 0.003) and VEGF (*p* = 0.01). Data are depictured as median with 25–75% percentiles and minimum—maximum and evaluated using the Mann–Whitney *U* test. Statistical significance was set at *p* < 0.05

Eotaxin/CCL11 (25.5, 10.4–62.2 vs 12.5, 3.8–56.4; *p* = 0.001), GM-CSF (10.4, 3.1–25.1 vs 6.9, 0.0–26.1; *p* = 0.01), GRO/CXCL1 (69.7; 0.0–895.6 vs 9.7, 0.0–249.0; *p* = 0.041), IL-10 (7.7, 0.0–41.3 vs 0.0, 0.0–5.2; *p* = 0.003), sCD40L (24.2, 0.0–226.5 vs 5.5, 0.0–229.2; *p* = 0.001), IL-1RA (23.5, 0.0–93.5 vs 0.0, 0.0–36.6; *p* = 0.0005), IL-1α (8.6, 0.0–55.9 vs 0.0, 0.0–31.2; *p* = 0.003) and VEGF (382.5, 0.0–1078.0 vs 200.8, 63.1–461.5; *p* = 0.01) levels have been increased in RA individuals (Fig. 2).

## Discussion

In our study, we have observed numerous alterations of cytokines and chemokines’ secretion between low disease activity RA and OA subjects with few of them attracting greater attention.

IL-2 is a Th-1 cytokine required for: activation, proliferation, differentiation and survival of lymphocytes with special regard to regulatory T cells (Tregs). It is also necessary for Tregs stability in peripheral tissues. The protein is mainly produced by activated helper T cells, but the ability to secrete it is also shared by activated cytotoxic T cells, natural killer cells or dendritic cells. Disturbed IL-2 pathway has been described in various autoimmune disorders [13], e.g., RA sufferers display heightened titer of anti-IL-2 antibodies that may affect cytokine bioavailability for Tregs [14]. Our results indicate increased IL-2 concentration in serum samples and more frequent protein detection in synovial fluid samples (data not shown) isolated from RA subjects with low disease activity when compared to OA group. Increased cytokine plasma/serum levels have been described before RA symptoms manifestation, during disease onset [15] as well as in already established disease [16]. Raza et al. detected high IL-2 concentrations in synovial fluids of newly diagnosed patients, but those levels significantly dropped as RA progressed [17]. Our study confirmed high levels of IL-2 as characteristic for sufferers with established RA even with DAS28 score ≤ 3.2.

C–C motif ligand 11 chemokine (CCL11), also described as eotaxin, is a ligand for C–C chemokine receptor 3 (CCR3) with the capacity to bind to other receptors as well [18]. CCL11 is secreted by: endothelial and epithelial cells, eosinophils, fibroblasts, keratinocytes, smooth muscle cells or chondrocytes [19], and its expression is induced by TNF and IL-4 [20]. It affects migration and activation of various leukocytes including: mast cells, eosinophils, Th2 lymphocytes, basophils, neutrophils and macrophages [18, 19, 21]. CCL11 and CCR3 production was proven in synovial tissue and synoviocytes of RA-affected joints, with the expression being stimulated by TNF and CCL11 itself. It was also confirmed that chemokine promotes synoviocytes migration during RA [18]. CCL11/CCR3 axis is also believed to affect bone destruction [19]. Surprisingly, Syversen et al. distinguished increased serum CCL11 levels as a predictor of slower radiographic progression of early RA [22]. We have found elevated chemokine concentration in serum and synovial fluid of our RA group when compared to OA counterparts, which further indicates involvement of this protein in RA-induced joint inflammation. We have observed significant correlation between synovial fluid concentrations of CCL11 and TNFα, but not IL-4 (data not shown). Similar results have been published by Wakabayashi et al. when comparing protein serum levels between newly diagnosed RA sufferers and healthy controls as well as synovial fluid concentrations between RA group and OA subjects. Authors also revealed correlation between synovial fluid quantities of CCL11 and TNF and concluded chemokines' responsibility for destructive activity of synoviocytes [18]. Increased plasma CCL11 concentration in newly diagnosed RA patients was also described by Kokkonen et al. Group even indicated it as one of the most important factors that shows altered expression due to RA onset [15]. Analyses conducted on long-standing RA patients have also shown increase in CCL11 serum concentration when compared to healthy volunteers along with its correlation with TNF levels. However, in referred study, chemokine concentrations were highest in synovial fluid samples [21], which is not convergent with our observations.

C–X–C motif chemokine ligand 1 (CXCL1)/GRO is a chemotactic cytokine engaged in the development of many inflammatory disorders, although its significance has been most extensively evaluated in tumors. CXCL1 serves as potent regulator of: neutrophil recruitment, angiogenesis and wound healing [23, 24]. Its receptors include, inter alia, CXCR2 (CXC motifs receptors 2) that is mainly expressed on neutrophils, but can be also detected on: monocytes, NK cells, mast cells, basophils, cytotoxic T and endothelium. In the course of RA, synovial macrophages, recognized as main source of chemokine, induce CXCL1-dependent neutrophil migration into the joint, where cells become activated and produce further factors to amplify their own recruitment and stimulation [25]. CXCL1 could also be responsible for hypertrophy and apoptosis of chondrocytes [26]. Sadik et al., utilizing murine model, indicated direct IL-17-induced secretion of CXCL1 by synovial fibroblasts during effector phase of arthritis [27]. Our results signify increased concentrations of chemokine in blood sera as well as synovial fluids of low disease activity RA patients when compared to OA counterparts. Similar results have been published by Koch et al. with authors indicating RA synovial tissue-isolated neutrophils as additional source of chemokine [28]. Flow cytometry and RT-PCR analyses revealed enhanced CXCL1 expression in chondrocytes isolated from cartilages of RA and OA patients when compared to healthy subjects [29]. Increased CXCL1 mRNA expression and protein production in synovial fibroblasts isolated from RA and OA sufferers when contrasted with healthy donors cells have also been described [24]. CXCL1 concentrations in RA and OA bone marrow plasma have been found similar [30].

Another chemokine that brought our attention was C–C motif ligand 22 chemokine (CCL22) also known as macrophage-derived chemokine (MDC)—protein produced by macrophages, dendritic cells or osteoclasts. It selectively binds to the C–C chemokine receptor 4 (CCR4) that is expressed by Th2, Th17 and Tregs cells [31]. CCL22 is further described as attenuating the development and function of Tregs by inhibiting the expression of Foxp3 transcription factor in STAT5-dependent manner [32]. Its role in inflammation could be considered quite ambiguous, since this potent chemotactic molecule affects both anti- and pro-inflammatory leukocytes [33]. It was also implied that CCL22, through pro-apoptotic activity on chondrocytes, takes direct part in initiating cartilage degeneration in OA—chemokine was appointed biomarker of cartilage degeneration in OA [34] and potential biomarker of early OA [35]. Surprisingly, CCL22 serum levels were lowered in our RA patients when compared to OA volunteers, but the differences in protein concentrations in synovial fluid samples did not reach statistical significance. Numerous publications reported increase in CCL22 sera levels of RA sufferers when contrasted with healthy [31, 32] or OA [31] individuals. Similar results were published concerning synovial fluid samples [31, 36]. Immunohistochemical analyses also revealed different distribution of CCL22 + cells in synovial tissue of RA and OA subjects [31]. Rump et al. did not find differences in chemokine concentration between RA and OA groups when analysing serum and synovial fluid samples, but reported more common expression of CCL22 in endothelial cells of synovium vessels in RA patients when compared to non-RA individuals. Authors concluded that this increased chemokine production may be responsible for augmented leukocytes recruitment to RA synovium [37]. Our results could be considered contradictory to those studies, since they suggest greater systemic production of CCL22 in OA patients and comparable protein levels in joint tissue. Although we are unable to pinpoint cause of differences between ours and others results, we would like to emphasize more frequent hypertension occurrence in OA group. To the best of our knowledge, there are no publications concerning CCL22 levels in hypertensive patients, but murine model analyses performed by Zhong et al. suggest increased chemokine secretion due to disease development [38]. Ren et al. described increased protein expression in articular chondrocytes during OA-induced cartilage damage [34]. The following studies of this group also revealed: heightened synovial fluid chemokine concentration in OA group when compared to healthy subjects, its correlation with synovitis and constricting impact of CCL22 on anti-inflammatory cytokines expression (33).

We also observed elevated concentration of bone-marrow-derived factors including granulocyte–macrophage colony-stimulating factor (GM-CSF). Numerous cells have been described as GM-CSF producers: fibroblasts, synoviocytes, innate lymphoid cells, endothelial cells and activated T lymphocytes. Its secretion is stimulated by IL-17 and IL-2. GM-CSF is indicated as a main cytokine responsible for maintaining RA-induced joint inflammation. Synoviocytes, lymphocytes and innate lymphoid cells initiate inflammatory cascade in GM-CSF-dependent manner that leads to monocyte/macrophage activation and severe release of: IL-1, IL-6 and TNF. These cytokines, among other things, are responsible for bone destruction [39]. In our study, both serum and synovial fluid concentrations of GM-CSF were heightened in low disease activity RA sufferers when compared to OA subjects. Similar results were described numerous times including patients suffering from moderate to severe forms of the disease [40, 41]. On the other hand, Raza et al. described elevated quantities of synovial fluid GM-CSF only in early diagnosed patients [17]. Continuously heightened GM-CSF serum and synovial fluid levels that have been observed by our group support the concept of protein being promising therapeutic target in RA as indicated by others [41].

We also noticed heightened levels of angiogenesis related factors including vascular endothelial growth factor (VEGF). VEGF is a pro-angiogenic protein—it promotes new blood vessels formation by, inter alia, stimulating proliferation and migration of endothelial cells. VEGF production is induced by hypoxic conditions and some cytokines. Growth factor also promotes inflammation maintenance in various conditions including RA. VEGF is considered responsible for synovial hypertrophy, swelling or cartilage and bone degeneration in RA [42]. We have detected increased growth factor concentration in serum as well as synovial fluid samples of RA patients when contrasted with OA volunteers. Kokkonen et al. reported raised growth factor serum levels in newly diagnosed patient. Group even indicated this protein crucial factor that discriminates between individuals before disease onset and patients with already established RA [15]. Results similar to ours were presented by Raza et al. when describing VEGF synovial fluid levels—highest concentration was characteristic for newly diagnosed individuals, but significant differences were also recognized between long-standing patients and OA controls [17]. Studies showed not only increased concentration of VEGF in serum and synovial fluid in RA patients [43], but also its correlation with inflammation and joint destruction markers [44]. Alterations of VEGF serum concentrations have been demonstrated a valuable response to treatment indicator [45], which is consistent with the fact that our RA patients, despite the treatment, failed to reach remission.

IL-10 is well-characterized immunomodulatory cytokine that affects innate and adaptive immune responses. It inhibits, inter alia, nuclear factor kappa B (NF-κB) pathway—this leads to suppression of various pro-inflammatory cytokines, including the ones strongly associated with arthritis [46, 47]. IL-10 affects production of: IL-2, IL-5, IFN-γ, TNFα or GM-CSF by CD4 + T cells. Cytokine is also recognized for decreasing MHC class II complex expression (48). IL-10 is produced by activated lymphocytes, monocytes and macrophages [48, 49], and its production by T helper cells is described as critical self-regulation mechanism [48]. IL-10 has been detected in synovial membrane and fluids of RA individuals—it is possible that cytokine is powerful enough to control acute inflammation, but becomes inadequate in case of constant stimulation that accompanies arthritis [49]. Anti-inflammatory properties of IL-10 have been well documented, but generic effect of its activity is context-dependent—e.g., simultaneous IL-2 and IL-10 stimulation increases the cytotoxicity of CD8 + T lymphocytes [48]. IL-10 also promotes: proliferation, survival and immunoglobulin production by B lymphocytes [47]. We have detected higher IL-10 concentrations in synovial fluid samples of low disease activity RA patients when contrasted with OA volunteers—in the latter case, protein was hardly ever detected. Cytokine was also undetectable in blood samples. Lettesjö et al. have also detected higher concentrations of IL-10 in synovial fluids of RA patients when compared to individuals with other arthritic lesions with protein often failing to reach detection limit in the second group [50]. Hernández-Bello et al., investigating role of genetic factors on IL-10 expression in moderate disease activity RA, observed heightened expression of cytokine mRNA in patients’ leukocytes when compared to cells isolated from healthy subjects, but did not detect significant differences when analysing serum protein levels (*p* = 0.2) [51]. On the other hand, Tukaj et al. detected increased IL-10 serum levels in moderate disease activity RA patients [52]. Elevated cytokine levels were also found in sera and synovial fluids of RA patients by Cush et al. [53]. Saxena et al. suggested that increased IL-10 secretion in RA individuals could simultaneously suppress cytokines’ production and cellular responses as well as intensify humoral autoimmune reactions (47).

IL-1RA is a natural endogenous IL-1 receptor type 1 (IL-1R1) antagonist, and therefore, it is considered antagonist of both—IL-1α and IL-1β signaling. Interestingly, binding affinity of IL-1RA and cytokines to the receptor is similar, enabling efficient suppression IL-1R1-mediated signaling by the antagonist. IL-1RA is described as essential in tissue repair and regeneration in IL-1α- and IL-1β-based pathologies. Since cartilage destruction in RA is primarily caused by IL-1β, the importance of IL-1RA in synovial inflammation suppression could be deemed invaluable [54, 55]. Numerous studies have proved attenuation of disease symptoms due to antagonist administration [56]. Moreover, mice with IL-1RA knockout develop RA-like disease and are utilized as RA model [57]. IL-1RA serum levels in our RA patients were higher when contrasted with OA sufferers, but observed differences did not reach statistical significance. However, our low disease activity RA group was characterized by higher IL-1RA as well as IL-1α concentrations in synovial fluid samples, with proteins being rarely detected in OA volunteers. Observed differences could be, at least partially, explained by therapeutic interventions—majority of our RA patients have been treated with DMARDs and/or steroids with such therapies being described as promoting IL-1RA secretion [58]. Increased cytokine secretion could also result from more pronounced joint destruction in our RA subject when compared to OA group and/or fairly unavoidable disease progression—significantly higher IL-1RA levels have been observed in destructive RA arthritis patients when contrasted with non-destructive arthritis [59]. Observed alterations in IL-1RA synovial fluid levels could also constitute response to heightened IL-1α concentrations—we have observed moderate (*R* = 0.45) correlation between both cytokines in group consisting of RA and OA patients (data not shown).

## Limitations of the study

The greatest limitation of our study is low number of participants. Individuals suffering from RA were significantly younger and had lower BMI scores, although it has been reported that RA patients undergo knee arthroplasty younger than their OA counterparts [60]. All of our RA patients have already received standard treatment which may have had significant impact on our results. OA volunteers more frequently suffered from arterial hypertension. Our conclusions are rather of a speculative nature and will require further analyses conducted on larger population as well as patients in different stages of RA progression.

## Conclusion

Development of the RA is a complex process that involves interactions between immune cells and numerous cytokines/chemokines. These factors, by affecting subsequent cell populations, are responsible for local and systemic symptoms of RA. We have observed alterations in blood serum and synovial fluid concentrations of many cytokines and chemokines, out of which: IL-2, eotaxin/CCL11, MDC/CCL22, GM-CSF, VEGF, IL-10 and IL-1RA have caught our attention. The unexpected decrease of MDC/CCL22 serum concentration in RA patients is particularly interesting and could be considered target of more thorough investigation. Our results considering anti-inflammatory cytokines may indicate continuous attempt to restore local cytokine homeostasis in low disease activity RA patients. However, increased concentrations of numerous pro-inflammatory factors as well as inability to achieve remission suggest insufficiency of those mechanisms even in a case of good RA management. Distinguished factors could serve as possible indicators of disease progression even in low disease activity RA patients.

## Data Availability

Data underlying this article will be shared on reasonable request to the corresponding author.
